# Quorum Sensing Extracellular Death Peptides Enhance the Endoribonucleolytic Activities of *Mycobacterium tuberculosis* MazF Toxins

**DOI:** 10.1128/mBio.00685-18

**Published:** 2018-05-01

**Authors:** Akanksha Nigam, Sathish Kumar, Hanna Engelberg-Kulka

**Affiliations:** a Department of Microbiology and Molecular Genetics, IMRIC, the Hebrew University-Hadassah Medical School, Jerusalem, Israel; University of Washington

**Keywords:** extracellular death peptides, *Mycobacterium tuberculosis*, quorum sensing

## Abstract

*mazEF* is a toxin-antitoxin module located on chromosomes of most bacteria. MazF toxins are endoribonucleases antagonized by MazE antitoxins. Previously, we characterized several quorum sensing peptides called "extracellular death factors" (EDFs). When secreted from bacterial cultures, EDFs induce interspecies cell death. EDFs also enhance the endoribonucleolytic activity of Escherichia coli MazF. Mycobacterium tuberculosis carries several *mazEF* modules. Among them, the endoribonucleolytic activities of MazF proteins mt-1, mt-3, and mt-6 were identified. MazF-mt6 and MazF-mt-3 cleave M. tuberculosis rRNAs. Here we report the *in vitro* effects of EDFs on the endoribonucleolytic activities of M. tuberculosis MazFs. Escherichia coli EDF (*Ec*EDF) and the three Pseudomonas aeruginosa EDFs (*Pa*EDFs) individually enhance the endoribonucleolytic activities of MazF-mt6 and MazF-mt3 and overcome the inhibitory effect of MazE-mt3 or MazE-mt6 on the endoribonucleolytic activities of the respective toxins. We propose that these EDFs can serve as a basis for a novel class of antibiotics against M. tuberculosis.

## IMPORTANCE

Mycobacterium tuberculosis is one of the leading causes of death from infectious disease. M. tuberculosis is highly drug resistant, and drug delivery to the infected site is very difficult. In previous studies, we showed that extracellular death factors (EDFs) can work as quorum sensing molecules which participate in interspecies bacterial cell death. In this study, we demonstrated the role of different EDFs in the endoribonucleolytic activities of M. tuberculosis MazFs. Escherichia coli EDF (*Ec*EDF) and the three Pseudomonas aeruginosa EDFs (*Pa*EDFs) individually enhance the endoribonucleolytic activities of MazF-mt6 and MazF-mt3. The current report provides a basis for the use of the EDF peptides *Ec*EDF and *Pa*EDF as novel antibiotics against M. tuberculosis.

## INTRODUCTION

Toxin-antitoxin (TA) modules are abundant on the chromosomes of most bacteria (1–8), including pathogens such as Staphylococcus aureus (7) and Mycobacterium tuberculosis, which carries at least 88 putative TA systems (8). Each of these modules consists of a pair of genes, usually transcribed as operons, in which the downstream gene encodes a stable toxin and the upstream gene encodes an unstable antitoxin (1–8). Among them, the most extensively studied and the first to have been described is the Escherichia coli *mazEF* system (9). E. coli MazF functions as a stable toxin, and MazE functions as an antitoxin degraded by ClpPA protease (9). E. coli MazF toxin is a sequence-specific endoribonuclease cleaving at ACA site (10) that was initially described to preferentially cleave single-stranded mRNAs and therefore was designated an mRNA interferase (11). However, subsequently, E. coli MazF was also shown to target the 16S rRNA within the 30S ribosomal subunit at the decoding center, thereby removing the anti-Shine-Dalgarno (aSD) sequence required for the initiation of the translation of canonical mRNAs (12). Moreover, the term “mRNA interferase” was also challenged by Woychik and colleagues (13, 14), who characterized the modes of action of two MazFs found in M. tuberculosis, MazF-mt6 and MazF-mt3. They identified these two mycobacterial toxins as endoribonucleases that cleave not only mRNAs but also rRNAs found inside the ribosomes (13, 14). Recently, the structures of M. tuberculosis MazF4 and MazF-mt6 were identified (15, 16). The study of MazF4 demonstrates the interaction of MazF4 with its RNA substrate and also with a new example of homology, extracellular death factors (EDFs) from M. tuberculosis (15). Moreover, the results from the studies by Woychik and colleagues show that MazF-mt6 not only cleaves at UU↓CCU sites of mRNAs but also cleaves the M. tuberculosis 23S rRNA at its single UU↓CCU site (13), which is located in the evolutionarily conserved helix/loop of domain IV that facilitates several critical ribosomal functions (13). By cleaving this crucial site of the ribosomal 23S rRNA, MazF-mt6 inhibits protein synthesis. In this way, the action of MazF-mt6 probably leads to bacterial growth arrest and to the generation of dormant M. tuberculosis cells (13). In contrast to MazF-mt6, which cleaves at UU↓CCU, Woychik and colleagues (14) discovered that M. tuberculosis MazF-mt3 cleaves at the U↓CCUU sequence, which is found in both the 23S and the 16S ribosomal subunits. By cleaving both subunits, MazF-mt3 can inactivate not just one but two critical components of the M. tuberculosis ribosomes (14). Like MazF-mt6, MazF-mt3 targets the essential, evolutionarily conserved helix/loop 70 of 23S rRNA. However, unlike MazF-mt6, MazF-mt3 also targets the anti-Shine-Dalgarno (aSD) sequence of 16S rRNA (14). Thus, we see that, perhaps even more than MazF-mt6, MazF-mt3 is involved in inhibiting protein synthesis and probably in growth arrest and in the generation of dormant M. tuberculosis cells (14).

Recently, we characterized several quorum sensing peptides that we called extracellular death factors (EDFs) (17, 18). EDFs secreted from bacterial cultures induce interspecies cell death (18). E. coli secretes E. coli EDF (*Ec*EDF), the NNWNN pentapeptide (5 amino acids) (17). Bacillus subtilis secretes B. subtilis (*Bs*EDF), the RGQQNE hexapeptide (6 amino acids) (18). Pseudomonas aeruginosa secretes three EDFs: P. aeruginosa EDF-1 (*Pa*EDF-1), the INEQTVVTK nonapeptide (9 amino acids); *Pa*EDF-2, the VEVSDDGSGGNTSLSQ hexadecapeptide (16 amino acids); and *Pa*EDF-3, the APKLSDGAAAGYVTKA hexadecapeptide (18). When we studied the effects of each of these EDF peptides on E. coli MazF, we found that, *in vitro*, though their sequences differ, each one significantly amplifies the endoribonucleolytic activities of E. coli MazF (18, 19). Here, we asked whether each of these different EDFs (including *Ec*EDF, *Bs*EDF, and all three *Pa*EDFs) might also enhance the endoribonucleolytic activities of the various M. tuberculosis MazF toxins as we showed in previous studies with E. coli MazF (18, 19). If so, these EDFs may serve as a basis for a bacteriostatic or even bactericidal effect on M. tuberculosis.

## RESULTS

### *Ec*EDF enhances the *in vitro* endoribonucleolytic activity of the M. tuberculosis toxin MazF-mt6.

**FIG 1:**
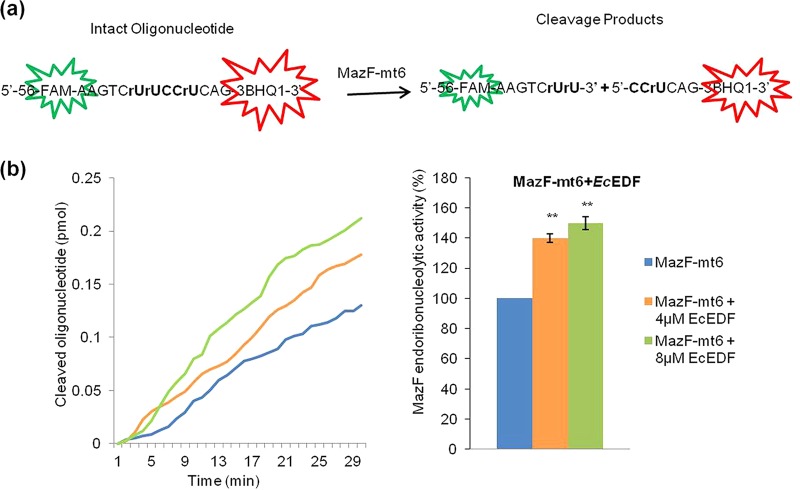
*Ec*EDF amplified the *in vitro* endoribonucleolytic activity of MazF-mt6. (a) Illustration of the reaction used for studies on the endoribonucleolytic activity of MazF-mt6. Cleavage of the chimeric ﬂuorescent oligonucleotide by MazF was measured as an increase of ﬂuorescence emission of the ﬂuorophore FAM. The cleavage site was UU↓CCU; “r” represents an RNA base. (b) (Left) Addition of (4 µM or 8 µM) wild-type *Ec*EDF (NNWNN) led to an increase in the *in vitro* activity of MazF-mt6. (Right) The relative (percent) increase of MazF-mt6 activity caused by the addition of *Ec*EDF. MazF-mt6 activity without the addition of *Ec*EDF was defined as 100%. The data represent means ± standard deviations of results from three experiments performed in triplicate. *, *P* < 0.01; **, *P* < 0.001; ***, *P* < 0.0001 (statistical significance compared to the control data).

We started by studying the effects of each of the several EDF peptides on the endoribonucleolytic activity of the M. tuberculosis MazF toxin MazF-mt6. Using affinity chromatography, as we have described previously (19), we prepared highly purified MazF-mt6.To measure MazF-mt6 activity, we used the continuous fluorometric assay (20) for the quantification and kinetic analysis of MazF endoribonucleolytic activity in real time. As mentioned above, the MazF-mt6 target site is UU↓CCU (13). Therefore, as a substrate for MazF-mt6, we used a chimeric oligonucleotide composed of RNA bases (rU), including UU↓CCU, flanked by DNA nucleotides, and labeled with a fluorophore molecule (6-carboxyfluorescein [FAM]) at its 5′ end and a quencher molecule (BHQ1) at its 3′ end. Cleavage of this constructed substrate by MazF-mt6 led to an increase in the distance between the fluorophore and the quencher, causing a significant increase in the fluorescence signal corresponding to FAM (Fig. 1a). Adding *Ec*EDF to this reaction mixture led to an increase in MazF-mt6 activity. Adding 4 µM *Ec*EDF led to an increase in MazF-mt6 activity of 40%, and adding 8 µM *Ec*EDF led to an increase of 55% (Fig. 1b).

### The ability of *Ec*EDF to enhance the *in vitro* activity of MazF-mt6 was dependent on the specific amino acid sequence of the *Ec*EDF molecule.

**FIG 2:**
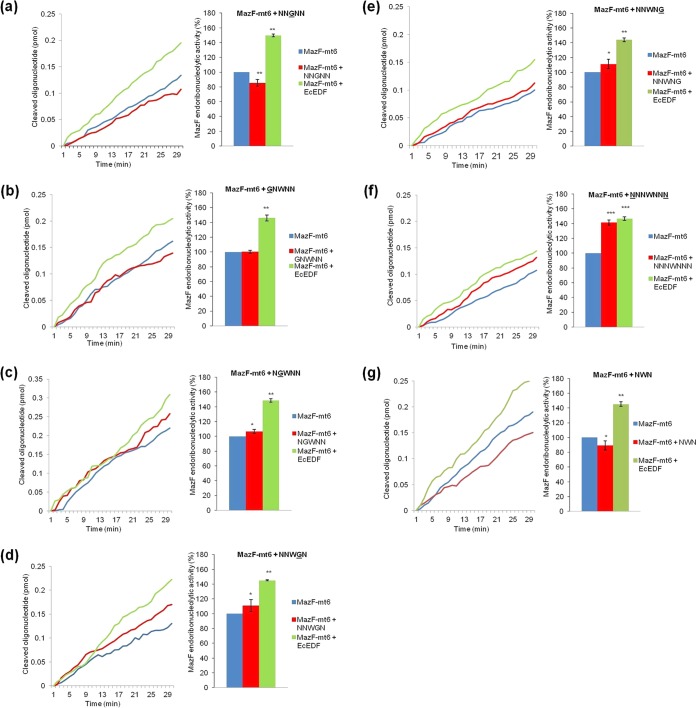
The importance of *Ec*EDF sequence for increasing MazF-mt6 endoribonucleolytic activity *in vitro*. (a) The third residue of *Ec*EDF, tryptophan (W), was replaced with glycine (G) as follows: NNGNN. (b) The first residue of *Ec*EDF, asparagine (N), was replaced with (G) as follows: GNWNN. (c) The second residue of *Ec*EDF, (N), was replaced with (G) as follows: NGWNN. (d) The fourth residue of *Ec*EDF, (N), was replaced with (G) as follows: NNWGN. (e) The fifth residue of *Ec*EDF, (N), was replaced with (G) as follows: NNWNG. (f) Wild-type (Wt) *Ec*EDF was lengthened by the addition of a new “N” residue at each end as follows: NNNWNNN. (g) Wt *Ec*EDF was shortened by the removal of both external “N” residues at each end as follows: NWN. The data represent means ± standard deviations of results from three experiments performed in triplicate. *, *P* < 0.01; **, *P* < 0.001; ***, *P* < 0.0001 (statistical significance compared to the control data).

We wondered about the contribution to the enhancement of MazF-mt6 activity of each of the amino acids in the pentapeptide *Ec*EDF. We constructed and tested seven derivatives of NNWNN, the wild-type *Ec*EDF, in which we either replaced each of the original amino acids with a glycine residue (G) or changed the length of the peptide. Replacing each of the amino acids of the wild-type sequence significantly interfered with the ability of *Ec*EDF to enhance the *in vitro* activity of MazF-mt6 (Fig. 2a to e). Replacing the central tryptophan (W) with glycine (G) (Fig. 2a) or removing the asparagine residues (N) from each end (NWN) (Fig. 2g) eliminated the peptide-induced stimulation of MazF-mt6 which had been observed with the native peptide. Adding N residues at each end to create the *Ec*EDF septapeptide NNNWNNN did not significantly interfere with the ability of *Ec*EDF to enhance MazF-mt6 activity (Fig. 2f).

### *Ec*EDF overcomes the inhibitory effect of MazE-mt6 on the endoribonucleolytic activity of MazF-mt6.

**FIG 3:**
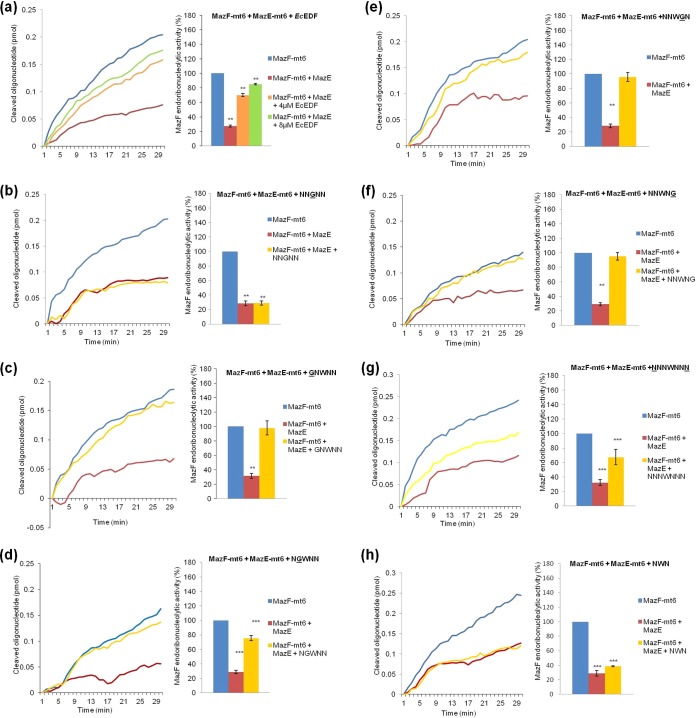
*Ec*EDF overcomes the inhibitory effect of MazE-mt6 on MazF-mt6 endoribonucleolytic activity *in vitro*. (a) A mixture of (0.5 µM) MazE-mt6 and (4 µM or 8 µM) *Ec*EDF (NNWNN) was added to preparations of (0.5 µM) MazF-mt6. (Left) The *in vitro* activity of MazF-mt6 was inhibited in the presence of MazE-mt6. (Right) Percent (%) increase in MazF-mt6 activity after the addition of *Ec*EDF to wells containing both MazF-mt6 and MazE-mt6. (b) Data were determined as described for panel a, but instead of NNWNN, NNGNN was added. (c) Data were determined as described for panel a, but instead of NNWNN, GNWNN was added. (d) Data were determined as described for panel a, but instead of NNWNN, NGWNN was added. (e) Data were determined as described for panel a, but instead of NNWNN, NNWGN was added. (f) Data were determined as described for panel a, but instead of NNWNN, NNWNG was added. (g) Data were determined as described for panel a, but instead of NNWNN, a lengthened, modified EDF to which a new N residue was attached at each end (NNNWNNN) was added. (h) Data were determined as described for panel a, but instead of NNWNN, a shortened, modified EDF from which an N residue has been removed from each end (NWN) was added. The data represent means ± standard deviations of results from three experiments performed in triplicate. *, *P* < 0.01; **, *P* < 0.001; ***, *P* < 0.0001 (statistical significance compared to the control data).

We also asked whether *Ec*EDF could overcome the inhibitory effect of the antitoxin MazE-mt6 on the endoribonucleolytic activity of MazF-mt6. To this end, we carried out an experiment under conditions in which MazE-mt6 almost completely inhibited MazF-mt6 activity. As shown, this inhibitory effect was almost completely reversed by adding *Ec*EDF (Fig. 3a). We also tested the importance of each of the amino acids of *Ec*EDF in overcoming the inhibitory effect of MazE-mt6. We found that only the central amino acid, tryptophan, played a critical role. When tryptophan (W) was replaced with glycine (G), the *Ec*EDF derivative no longer overcame inhibition by MazE-mt6 (Fig. 3b). In contrast, replacing each of the other amino acids of *Ec*EDF still permitted the derivatives to overcome the inhibitory effect of MazE-mt6 (Fig. 3c to 3e). In addition, lengthening of *Ec*EDF to NNNWNNN partially overcame the inhibitory effect of MazE-mt6 (Fig. 3f), and shortening the length of *Ec*EDF to NWN did not permit the herein described overcoming effect (Fig. 3g; for a summary, see Table 1).

**TABLE 1:** The requirement of each amino acid in *Ec*EDF (NNWNN) for the enhancement of MazF-mt3 and MazF-mt6 endoribonucleolytic activities and in overcoming the effect seen after the inhibition of the MazFs by the respective MazEs^a^

Sequence of *Ec*EDF	Enhancement of effect of:		Overcoming of effect of:	
	MazF-mt3	MazF-mt6	MazE-mt3	MazE-mt6
GNWNN	+	−	+	+
NGWNN	−	−	+	+
NNGNN	−	−	−	−
NNWGN	−	−	+	+
NNWNG	−	−	+	+

^a^ The data for MazF-mt6 were taken from Fig. 2 and 3. The data for MazF-mt3 were taken from Fig. S2 and S3. “−” indicates that the amino acid was required. “+” indicates that the amino acid was not required.

### As in the case of MazEF-mt6, *Ec*EDF both enhanced the endoribonucleolytic activity of M. tuberculosis toxin MazF-mt3 and overcame the inhibitory effect of MazE-mt3 on MazF-mt3.

Like MazF-mt6, MazF-mt3 is an M. tuberculosis MazF endoribonuclease. While the target site for MazF-mt6 is UU↓CCU (13), the target site for MazF-mt3 is U↓CCUU (14). We asked whether *Ec*EDF would affect MazF-mt3 activity as it affected MazF-mt6 activity. As we did for MazF-mt6, we analyzed the activity of a highly purified preparation of MazF-mt3 using the continuous fluorometric assay (20). As in the case of MazF-mt6 (Fig. 1b), adding MazF-mt3 to the reaction mixture led to an increase in fluorescence (see Fig. S1b in the supplemental material). Adding 4 µM *Ec*EDF to this reaction mixture led to an ~30% increase in MazF-mt3 activity, and adding 8 µM *Ec*EDF led to an ~55% increase in MazF-mt3 activity; thus, as in the case of MazF-mt6, the increase in MazF-mt3 activity caused by the addition of *Ec*EDF was concentration dependent (Fig. S1b). Moreover, *Ec*EDF also overcome the inhibitory effect of MazE-mt3 on the endoribonucleolytic activity of MazF-mt3 (Fig. S3a). Thus, the addition of *Ec*EDF affected Maz*EF*-mt6 (Fig. 3a) and Maz*EF*-mt3 (Fig. S3a) similarly. Adding 8 µM *Ec*EDF to the reaction mixtures led to nearly identical responses of Maz*E*-mt6 and Maz*E*-mt3: in each case, the antitoxin function of the MazE molecules was almost completely overcome. Note that the only residue of *Ec*EDF that was certainly required for overcoming the inhibitory effects of both MazE-mt6 (Fig. 3b) and MazE-mt3 (Fig. S3b) was the central tryptophan residue (W). The activity of MazE-mt3 was not affected by altered *Ec*EDF when any of the other amino acid residues was replaced (Table 1; see also Fig. S3c to f). However, no such complete similarity between the effects of the EDF peptides on the MazEF-mt6 and MazEF mt-3 systems occurred regarding their enhancement effect on the endoribonucleolytic activity of the two MazF toxins. With the exception of the first asparagine, the presence of each amino acid residue of *Ec*EDF was required to enhance the effects of MazF-mt3 (Table 1; see also Fig. S2b). However, even the presence of that first asparagine residue of *Ec*EDF was required to enhance the effects of MazF-mt6 (Table 1; see also Fig. 2b). In addition, the activities of both MazF-mt6 and MazF-mt3 were enhanced when we lengthened *Ec*EDF (NNWNN) by adding an asparagine residue at each end (NNNWNNN) (Fig. 2f and S2f) but not when we shortened NNWNN to NWN (Fig. 2g and S2g).

10.1128/mBio.00685-18.2 FIG S1  *Ec*EDF amplifies *in vitro* MazF-mt3 endoribonucleolytic activity. (a) Illustration of the reaction used for studies on the endoribonucleolytic activity of MazF-mt3. Cleavage of the chimeric ﬂuorescent oligonucleotide by MazF was measured as an increase of ﬂuorescence emission of the ﬂuorophore FAM. The cleavage site was U↓CCUU; “r” represents an RNA base. (b) (Left) Addition of (4 µM or 8 µM) *Ec*EDF led to an increase in the *in vitro* activity of MazF-mt3. (Right) Relative (percent) increase of MazF-mt3 activity caused by the addition of (4 µM or 8 µM) *Ec*EDF. MazF-mt3 activity without the addition of *Ec*EDF was defined as 100%. The data represent means ± standard deviations of results from three experiments performed in triplicate. *, *P* < 0.01; **, *P* < 0.001; ***, *P* < 0.0001 (statistical significance compared to the control data). Download FIG S1, TIF file, 19.9 MB. Copyright © 2018 Nigam et al. 2018 Nigam et al. This content is distributed under the terms of the Creative Commons Attribution 4.0 International license.

10.1128/mBio.00685-18.3 FIG S2  The importance of *Ec*EDF sequence for increasing MazF-mt3 endoribonucleolytic activity *in vitro*. (a) The third residue of *Ec*EDF, tryptophan (W), was replaced with glycine (G) as follows: NNGNN. (b) The first residue of *Ec*EDF, asparagine (N), was replaced with G as follows: GNWNN. (c) The second residue of *Ec*EDF, (N), was replaced with (G) as follows: NGWNN. (d) The fourth residue of *Ec*EDF, (N), was replaced with (G) as follows: NNWGN. (e) The fifth residue of *Ec*EDF, (N), was replaced with (G) as follows: NNWNG. (f) Wt *Ec*EDF was lengthened by the addition of a new “N” residue at each end as follows: NNNWNNN. (g) Wt *Ec*EDF was shortened by the removal of both external “N” residues at each end as follows: NWN. Data represent means ± standard deviations of results from three experiments performed in triplicate. *, *P* < 0.01; **, *P* < 0.001; ***, *P* < 0.0001 (statistical significance compared to the control data). Download FIG S2, TIF file, 19.9 MB. Copyright © 2018 Nigam et al. 2018 Nigam et al. This content is distributed under the terms of the Creative Commons Attribution 4.0 International license.

10.1128/mBio.00685-18.4 FIG S3  *Ec*EDF overcomes the inhibitory effect of MazE-mt3 on MazF-mt3 endoribonucleolytic activity. (a) A mixture of (0.5 µM) MazE-mt3 and (4 µM or 8 µM) *Ec*EDF was added to preparations of (0.5 µM) MazF-mt3. (Left) The *in vitro* activity of MazF-mt3 was inhibited in the presence of MazE-mt3. (Right) Percent (%) increase in MazF-mt3 activity after the addition of *Ec*EDF to wells containing both MazF-mt3 and MazE-mt3. (b) Data were determined as described for panel a, but instead of NNWNN, NNGNN was added. (c) Data were determined as described for panel a, but instead of NNWNN, GNWNN was added. (d) Data were determined as described for panel a, but instead of NNWNN, NGWNN was added. (e) Data were determined as described for panel a, but instead of NNWNN, NNWGN was added. (f) Data were determined as described for panel a, but instead of NNWNN, NNWNG was added. (g) Data were determined as described for panel a, but instead of NNWNN, a lengthened, modified EDF to which a new N residue was attached at each end (NNNWNNN) was added. (h) Data were determined as described for panel a, but instead of NNWNN, a shortened, modified EDF from which an N residue was removed from each end (NWN) was added. The data represent means ± standard deviations of results from three experiments performed in triplicate. *, *P* < 0.01; **, *P* < 0.001; ***, *P* < 0.0001 (statistical significance compared to the control data). Download FIG S3, TIF file, 19.9 MB. Copyright © 2018 Nigam et al. 2018 Nigam et al. This content is distributed under the terms of the Creative Commons Attribution 4.0 International license.

### The effects of EDFs from P. aeruginosa (*Pa*EDFs) on the endoribonucleolytic activities of M. tuberculosis toxins MazF-mt6 and MazF-mt3.

**FIG 4:**
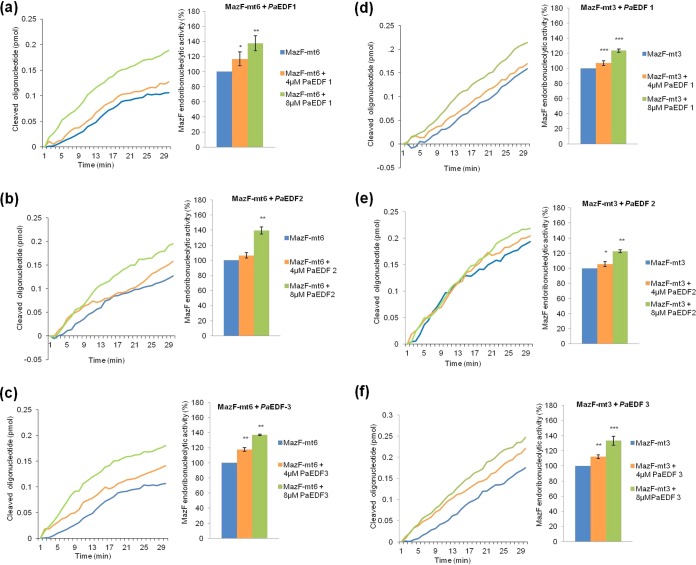
The effects of the addition of each of the EDFs of P. aeruginosa, i.e., *Pa*EDF-1, *Pa*EDF-2, or *Pa*EDF-3, on the endoribonucleolytic activities of M. tuberculosis MazF toxins MazF-mt6 and MazF-mt3. (a) (Left) *Pa*EDF-1 added to MazF-mt6. (Right) The relative (percent) increase of MazF-mt6 activity in the presence of *Pa*EDF-1. (b) (Left) *Pa*EDF-2 added to MazF-mt6. (Right) The relative (percent) increase of MazF-mt6 activity in the presence of *Pa*EDF-2. (c) (Left) *Pa*EDF-3 added to MazF-mt6. (Right) The relative (percent) increase of MazF-mt6 activity in the presence of *Pa*EDF-3. (d) (Left) Addition of *Pa*EDF-1 to MazF-mt3. (Right) The relative (percent) increase of MazF-mt3 activity in the presence of *Pa*EDF-1. (e) (Left) Addition of *Pa*EDF-2 to MazF-mt3. (Right) The relative (percent) increase of MazF-mt3 activity in the presence of *Pa*EDF-2. (f) (Left) Addition of *Pa*EDF-3 to MazF-mt3. (Right) The relative (percent) increase of MazF-mt3 activity in the presence of *Pa*EDF-3. MazF activity without the addition of EDF was defined as 100%. The data represent means ± standard deviations of results from three experiments performed in triplicate. *, *P* < 0.01; **, *P* < 0.001; ***, *P* < 0.0001 (statistical significance compared to the control data).

We also studied the effects of each of the three P. aeruginosa EDFs, *Pa*EDF-1, *Pa*EDF-2, and *Pa*EDF-3, on the M. tuberculosis MazF-mt6 and MazF-mt3 toxins. The addition of each of these *Pa*EDFs at a concentration of 4 µM or 8 µM led to an increase in the endoribonucleolytic activities of both MazF-m6 and MazF-mt3 (Fig. 4). The greatest increases in activity were caused by the addition of *Pa*EDF-3 (Fig. 4c and f); however, for both MazF-mt6 and MazF-mt3, those levels were lower than those caused by the addition of *Ec*EDF (Fig. 1; see also Fig. S1).

### *Pa*EDF overcomes the inhibitory effect of MazE-mt6 on the endoribonucleolytic activity of MazF-mt6 and the inhibitory effect of MazE-mt3 on the endoribonucleolytic activity of MazF-mt3.

**FIG 5:**
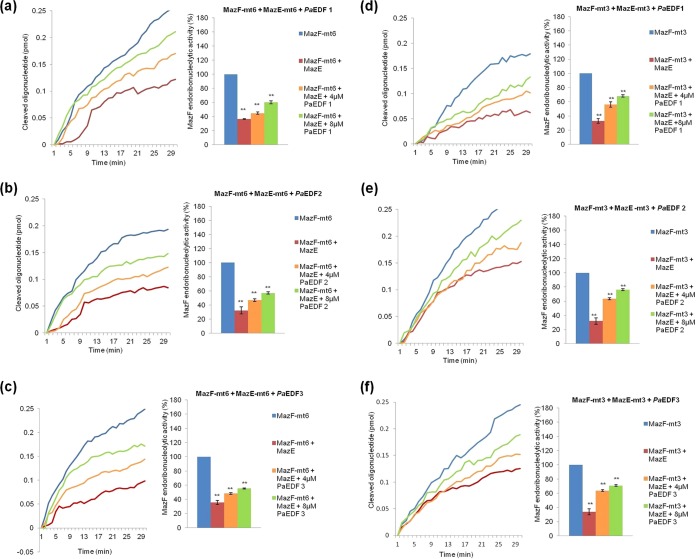
*Pa*EDFs partially overcomes the inhibitory effect of MazE-mt6 on MazF-mt6 and of MazE-mt3 on MazF-mt3 endoribonucleolytic activity *in vitro*. (a) A mixture of (0.5 µM) MazE-mt6 and (4 µM or 8 µM) *Pa*EDF-1 was added to preparations of (0.5 µM) MazF-mt6. (Left) The *in vitro* activity of MazF-mt6 was inhibited in the presence of MazE-mt6, and *Pa*EDF-1 overcomes this inhibitory effect. (Right) Percent (%) increase in MazF-mt6 activity after the addition of *Pa*EDF-1 to wells containing both MazF-mt6 and MazE-mt6. (b) Data were determined as described for panel a, but instead of *Pa*EDF-1, *Pa*EDF-2 was added. (c) Data were determined as described for panel a, but instead of *Pa*EDF-1, *Pa*EDF-3 was added. (d) A mixture of (0.5 µM) MazE-mt3 and (4 µM or 8 µM) *Pa*EDF-1 was added to preparations of (0.5 µM) MazF-mt3. (Left) The *in vitro* activity of MazF-mt3 was inhibitedin the presence of MazE-mt3 and *Pa*EDF-1 overcomes this inhibitory effect. (Right) Percent (%) increase in MazF-mt3 activity after the addition of *Pa*EDF-1 to wells containing both MazF-mt3and MazE-mt3. (e) Data were determined as described for panel d, but instead of *Pa*EDF-1, *Pa*EDF-2 was added. (f) Data were determined as described for panel d, but instead of *Pa*EDF-1, *Pa*EDF-3 was added. The data represent means ± standard deviations of results from three experiments performed in triplicate. *, *P* < 0.01; **, *P* < 0.001; ***, *P* < 0.0001 (statistical significance compared to the control data).

We also asked whether *Pa*EDF could overcome the inhibitory effect of the antitoxin MazE-6 on MazF-6. To this end, we carried out an experiment under conditions in which MazE almost completely inhibited MazF activity. As shown, this inhibitory effect was almost completely reversed by adding all three *Pa*EDFs (*Pa*EDF-1, *Pa*EDF-2, and *Pa*EDF-3) (Fig. 5a to c). Moreover, all three *Pa*EDFs also overcome the inhibitory effect of MazE-mt3 on the endoribonucleolytic activity of MazF-mt3 (Fig. 5d to f). Thus, the addition of *Pa*EDFs affected Maz*EF*-mt6 (Fig. 5a to c) and Maz*EF*-mt3 (Fig. 5d to f) similarly.

## DISCUSSION

Previously, we discovered extracellular death factors (EDFs) in several unrelated bacteria: *Ec*EDF in E. coli (17), *Bs*EDF in B. subtilis (18), and three *Pa*EDFs in P. aeruginosa (18). Of the many *mazEF* systems identified in M. tuberculosis so far, three, MazEF-mt6, MazEF-mt3, and MazEF-mt1, have been characterized and their cleaved sites identified (13, 14, 21). Here we studied how each of these various EDF peptides affected the *in vitro* activities of MazEF-mt6 (Fig. 1 to 5), MazEF-mt3 (see in the supplemental material), and MazEF-mt1 (Fig. S4). Our work (summarized in Table 2) revealed the following. (i) The *Ec*EDF peptide similarly activated two systems, MazF-mt3 and MazF-mt6. It enhanced the endoribonucleolytic activities of MazF-mt3 (Fig. S1b) and of MazF-mt6 (Fig. 1b); moreover, *Ec*EDF overcame the inhibitory effects of MazE-mt3 on the endoribonucleolytic activity of MazF-mt3 (Fig. S3a) and of MazE-mt6 on the endoribonucleolytic activity of MazF-mt6 (Fig. 3a). (ii) Unexpectedly, the exact sequence of wild-type *Ec*EDF (NNWNN) was more significant for enhancing the activities of the MazF toxins than for overcoming the effects of the MazE antitoxins (Table 1). Each of the wild-type amino acid residues was required to increase MazF-mt6 activity (Fig. 2) (Table 1), and, with the exception of the first asparagine residue, each of the wild-type amino acid residues was required to increase MazF-mt3 activity (see the left sides of the columns in Table 1; see also Fig. S2b). In contrast, only the central tryptophan residue of the wild-type *Ec*EDF was important for overcoming the effects of both the MazE-mt6 antitoxin (Fig. 3b) and the MazE-mt3 antitoxin (see the right sides of the columns in Table 1; see also Fig. S3b). (iii) In contrast to the effects of *Ec*EDF on the MazEF-mt6 and MazEF-mt3 toxin-antitoxin systems, not even one of the EDF peptides studied had any effect on the endoribonucleolytic activity of MazF-mt1 (Table 2; see also Fig. S4). (iv) The EDF from B. subtilis, *Bs*EDF, had no effect on either MazF-mt6 or MazF-mt3 (Table 2; see also Fig. S5 and Text S1 in the supplemental material). (v) Each of the *Pa*EDFs peptides affected the activities of both MazF-mt6 (Fig. 4a to c) and MazF-mt3 (Fig. 4d to f), though the effects were less than those seen with *Ec*EDF (Fig. 1b and Fig. S1b). In addition, none of the *Pa*EDFs affected the activity of MazF-mt1 (Table 2; see also Fig. S4c to e).

10.1128/mBio.00685-18.1 TEXT S1  Supplemental information. Download TEXT S1, DOCX file, 0.1 MB. Copyright © 2018 Nigam et al. 2018 Nigam et al. This content is distributed under the terms of the Creative Commons Attribution 4.0 International license.

10.1128/mBio.00685-18.5 FIG S4  *Ec*EDF did not amplify the *in vitro* endoribonucleolytic activity of MazF-mt1. (a) Illustration of the reaction used for studies on the endoribonucleolytic activity of M. tuberculosis MazF-mt1. Cleavage of the chimeric ﬂuorescent oligonucleotide by MazF was expressed as an increase of ﬂuorescence emission of the ﬂuorophore FAM. The cleavage site was U↓AC; “r” represents an RNA base. (b) (Left) Addition of (4 µM or 8 µM) *Ec*EDF did not increase the *in vitro* activity of (1.5 µM) MazF-mt1. (Right) Relative (percent) increase of MazF-mt1 activity caused by the addition of (4 µM or 8 µM) *Ec*EDF. (c) (Left) P. aeruginosa EDF-1 (*Pa*EDF-1) was added to M. tuberculosis MazF-mt1. (Right) The (%) change in the *in vitro* endoribonucleolytic activity of MazF-mt1 caused by the addition of *Pa*EDF-1. (d) (Left) P. aeruginosa EDF-2 (*Pa*EDF-2) was added to M. tuberculosis MazF-mt1. (Right) Relative (percent) changes in the *in vitro* endoribonucleolytic activity of MazF-mt1 caused by the addition of *Pa*EDF-2. (e) (Left) P. aeruginosa EDF-3 (*Pa*EDF-3) was added to M. tuberculosis MazF-mt1. (Right) Relative (percent) changes in the *in vitro* endoribonucleolytic activity of MazF-mt1 caused by the addition of *Pa*EDF-3. The activity of MazF-mt1 without the addition of any of the various EDF molecules was defined as 100%. The data represent means ± standard deviations of results from three experiments performed in triplicate. *, *P* < 0.01; **, *P* < 0.001; ***, *P* < 0.0001 (statistical significance compared to the control data). Download FIG S4, TIF file, 19.9 MB. Copyright © 2018 Nigam et al. 2018 Nigam et al. This content is distributed under the terms of the Creative Commons Attribution 4.0 International license.

10.1128/mBio.00685-18.6 FIG S5  Bacillus subtilis EDF (*Bs*EDF) does not affect the *in vitro* endoribonucleolytic activities of M. tuberculosis MazF-mt1, MazF-mt3, and MazF-mt6. (a) (Left) The effects of the addition of (4 µM or 8 µM) *Bs*EDF to M. tuberculosis MazF-mt1. (Right) Relative (percent) changes in the *in vitro* endoribonucleolytic activity of MazF-mt1 caused by the addition of (4 µM or 8 µM) *Bs*EDF. (b) (Left) The effects of the addition of (4 µM or 8 µM) *Bs*EDF to M. tuberculosis MazF-mt3. (Right) Relative (percent) changes in the *in vitro* endoribonucleolytic activity of MazF-mt3 caused by the addition of *Bs*EDF. (Left) *Bs*EDF (4 µM or 8 µM) was added to M. tuberculosis MazF-mt6. (Right) Relative (percent) changes in the *in vitro* endoribonucleolytic activity of MazF-mt6 caused by addition of *Bs*EDF. The activities of MazF-mt1, MazF-mt3, or MazF-mt6 without the addition of *Bs*EDF were defined as 100%. The data represent means ± standard deviations of results from three experiments performed in triplicate. *, *P* < 0.01; **, *P* < 0.001; ***, *P* < 0.0001 (statistical significance compared to the control data). Download FIG S5, TIF file, 19.9 MB. Copyright © 2018 Nigam et al. 2018 Nigam et al. This content is distributed under the terms of the Creative Commons Attribution 4.0 International license.

**TABLE 2:** The effect of each of the EDF peptides studied on the endoribonucleolytic activities of the M. tuberculosis toxins MazF-mt1, MazF-mt3, and MazF-mt6^a^

M. tuberculosis toxin	*Ec*EDF (NNWNN)	*Bs*EDF (RGQQNE)	*Pa*EDF-1 (INEQTVVTK)	*Pa*EDF-2 (VEVSDDGSGGNTSLSQ)	*Pa*EDF-3 (APKLSDGAAAGYVTKA)
MazF-mt1	−	−	−	−	−
MazF-mt3	+	−	±	±	+
MazF-mt6	+	−	±	±	+

^a^ “+” indicates a significant increase in the endoribonucleolytic activity of the EDF. “±” indicates a moderate increase in the endoribonucleolytic activity of the EDF. “−” indicates no effect on the endoribonucleolytic activity of the EDF.

Our results reported here on the effects of *Ec*EDF and of *Pa*EDF-1, *Pa*EDF-2, and *Pa*EDF-3 on the MazEF-mt6 and MazEF-mt3 toxin-antitoxin systems may be important in at least two ways. Clarifying the significance of each of the specific amino acid residues of *Ec*EDF required for enhancing the endoribonuclease activities of the MazF-mt6 and MazF-mt3 toxins and for overcoming the effects of the MazE-mt6 and MazE-mt3 antitoxins will surely contribute to our understanding of the structural elements of these two MazEF systems. Peptides from multiple organisms with different activities that can be modulated by changing specific amino acids represent a very complex but exciting set of molecular tools that can be exploited to study these structural elements. On a more practical level, our results may become the basis for developing the quorum sensing peptides *Ec*EDF and *Pa*EDF-1, *Pa*EDF-2, and *Pa*EDF-3 as a novel class of antibiotics against M. tuberculosis. Using these EDFs to increase the activities of MazF-mt6 and MazF-mt3 will lead to enhanced cleavage of the rRNAs in M. tuberculosis, causing inhibition of protein synthesis, (probably) growth arrest, and even cell death. According to a report from the World Health Organization (WHO), in 2016, M. tuberculosis infections caused 1.8 million deaths. Thus, our exciting results offer the promise of a solution to the global problem of a serious lack of appropriate antibiotics.

## MATERIALS AND METHODS

### Strains and plasmids.

We used E. coli strains BL21(DE3) (Invitrogen, Carlsbad, CA) and TG1 (our strain collection). We constructed plasmids pET28a-mazF-mt1(*His*)_6_, pET28a-mazF-mt3(*His*)_6_, and pET28a-mazF-mt6(*His*)_6_ from pET28a (Novagen, San Diego, CA) to express MazF(*His*)_6_ under the control of the T7 promoter. We also constructed plasmids pET28a-mazE-mt1(*His*)_6_, pET28a-mazE-mt3(*His*)_6_, and pET28a-mazE-mt6(*His*)_6_ from pET28a (Novagen, San Diego, CA) to express MazE(*His*)_6_.

### Synthetic oligonucleotides.

To study MazF-mt1 cleavage, we used an oligonucleotide with the sequence 5′-/5FAM/AAGTCrUACTCAG/3BHQ_1/-3′; for MazF-mt3 cleavage, we used an oligonucleotide with the sequence 5′-/5FAM/AAGTCrUCCrUrUCAG/3BHQ_1/-3′; and for MazF-mt6 cleavage, we used an oligonucleotide with the sequence 5′-/5FAM/AAGTCrUrUCCrUCAG/3BHQ_1/-3′. Here, “r” represents an RNA base. These oligonucleotides are labeled with 6-carboxyfluorescein (FAM) on the 5′ end and with black hole quencher-1 (BHQ_1) on the 3′ end (18), and the corresponding oligonucleotide cleavage fragments of MazF-mt1 (5′-/5FAM/AAGTCrU and ACTCAG/3BHQ_1/-3′), MazF-mt3 (5′-/5FAM/AAGTCrU and CCrUrUCAG/3BHQ_1/-3′), and MazF-mt6 (5′-/5FAM/AAGTCrUrU and CCrUCAG/3BHQ_1/-3′) were also used. These oligonucleotides were purchased from IDT.

### Production of (*His*)_6_ MazFs and (*His*)_6_ MazEs of M. tuberculosis.

To produce MazF(*His*)_6_, we transformed E. coli BL21(DE3) with pET28a-mazF-mt1(*His*)_6_ or pET28a-mazF-mt3(*His*)_6_ or pET28a-mazF-mt6(*His*)_6_. Transformants were grown overnight, and then the culture was diluted 1:50 in LB medium containing kanamycin (25 mg/ml) and 1 mM IPTG (isopropyl-β-d-thiogalactopyranoside). These cultures were then grown at 30°C to an optical density at 600 nm (OD_600_) of 0.5, and 1.0 mM IPTG was added, after which the bacteria were allowed to grow for an additional 2 h. Bacteria were harvested by centrifugation at 4,000 rpm at 4°C for 15 min. The bacterial pellets were stored at −80°C for no more than 2 weeks. To produce MazE(*His*)_6_, we transformed E. coli BL21(DE3) with pET28a-mazE-mt1(*His*)_6_ or pET28a-mazE-mt3(*His*)_6_ or pET28a-mazE-mt6(*His*)_6_. Transformants were grown overnight in LB medium as described above for MazF purification, except that we added 0.5 mM IPTG at an OD_600_ of 0.4, after which the bacteria were allowed to grow for an additional 2 h.

### Purification of MazF(*His*)_6_ and MazE(*His*)_6_ of M. tuberculosis*.*

We purified MazF(*His*)_6_ and MazE(*His*)_6_ at equal levels as follows. (i) Pellets of BL21(DE3) expressing either MazF(*His*)_6_ or MazE(*His*)_6_ were thawed at room temperature for 20 min and resuspended in 200 ml cold binding buffer (50 mM Tris-HCl, 300 mM NaCl, 10 mM imidazole, pH 8.0). Subsequent steps were performed at 4°C. (ii) Cells were incubated with lysozyme (0.25 mg/ml) for 30 min and then sonicated for 10 s three times at 30-s intervals. (iii) Lysates were centrifuged at 8,000 rpm for 30 min. (iv) To trap the proteins on resin, 4 ml nickel-nitrilotriacetic acid (Ni-NTA) resin (Adar Biotech, Rehovot, Israel) was mixed with the supernatant, and the mixture was then incubated with gentle shaking for 1 h. (v) Centrifugation was then performed at 8,000 rpm and 4°C for 10 min, and the resin was loaded with the protein on the column and left untreated for 10 min. (vi) The frit was added on the resin, and washing was started. (vii) The resin was washed with 20 ml binding buffer and then with 10 ml binding buffer containing 8 M urea, followed by seven additional washes using 10 ml binding buffer in which the concentration of urea was decreased by 1 M for each wash; finally, a wash was performed with 10 ml wash buffer (50 mM Tris-HCl, 300 mM NaCl, and 20 mM imidazole at pH 8.0). (viii) Elution of the proteins was performed using 5 ml elution buffer (50 mM Tris-HCl, 300 mM NaCl, and 250 mM imidazole at pH 8.0). (ix) The protein was collected in 10 aliquots, which were stored with 20% (final concentration) glycerol at −80^o^C. Protein concentrations were determined by using the Bradford assay (Bio-Rad, Germany).

### Determination of MazF activities by measuring the cleavage of fluorescent chimeric labeled substrate.

To determine MazF endoribonucleolytic activity quantitatively, we used a procedure previously described by Wang and Hergenrother (20). Wells of a black 96-well plate (Nunc, Thermo Fisher Scientific, Denmark) were filled with TE (10 mM Tris-HCI, 1 mM EDTA [pH 8.0]) and 0.5 µM labeled fluorescent oligonucleotide, and 0.5 µM MazF was added. Cleavage by MazF of the fluorescent oligonucleotide was measured by determination of an increase in FAM fluorescent emission (Fig. 1a). To study the effects on MazF activity of EDF (GenScript Corp., Piscataway, NJ) and its derivatives, we added to this reaction mixture 8 µM tested peptide or an equivalent volume of the peptide buffer as a control. In each well, we measured fluorescence using an excitation filter (485 ± 15 nm) and an emission filter (530 ± 15 nm) 30 times at intervals of 60 s for MazF-mt3 and MazF-mt6. On the other hand, we measured fluorescence for MazF-mt1 40 times at intervals of 60 s and considered the tenth cycle to represent the first cycle, because such reactions start at min 10 (Spark multiplate reader; Tecan). We assigned a value of 100% to the level of MazF activity seen in the absence of EDF. To determine the ratios of MazF activities in the presence or absence of EDF or mutated EDF, we calculated the slopes corresponding to the 15th and 25th readings of each reaction and compared those values to the 100% MazF value. We carried out experiments for each experimental peptide with various MazF productions at least three times; from the results of these experiments, we calculated the average activities and determined the standard deviations. The relative MazF activities are shown in the figures. A calibration plot in which fluorescence unit (FU) values were converted to product concentration values (expressed in picomoles) was constructed by the use of a 1:1 ratio of the synthetic oligonucleotide cleavage products.
