# Supplementary figures and images for: Quorum Sensing Extracellular Death Peptides Enhance the Endoribonucleolytic Activities of Mycobacterium tuberculosis MazF Toxins
Source: mBio. 2018 May 1;9(3):e00685-18. doi: 10.1128/mBio.00685-18 (PMC5930309; doi:10.1128/mBio.00685-18)

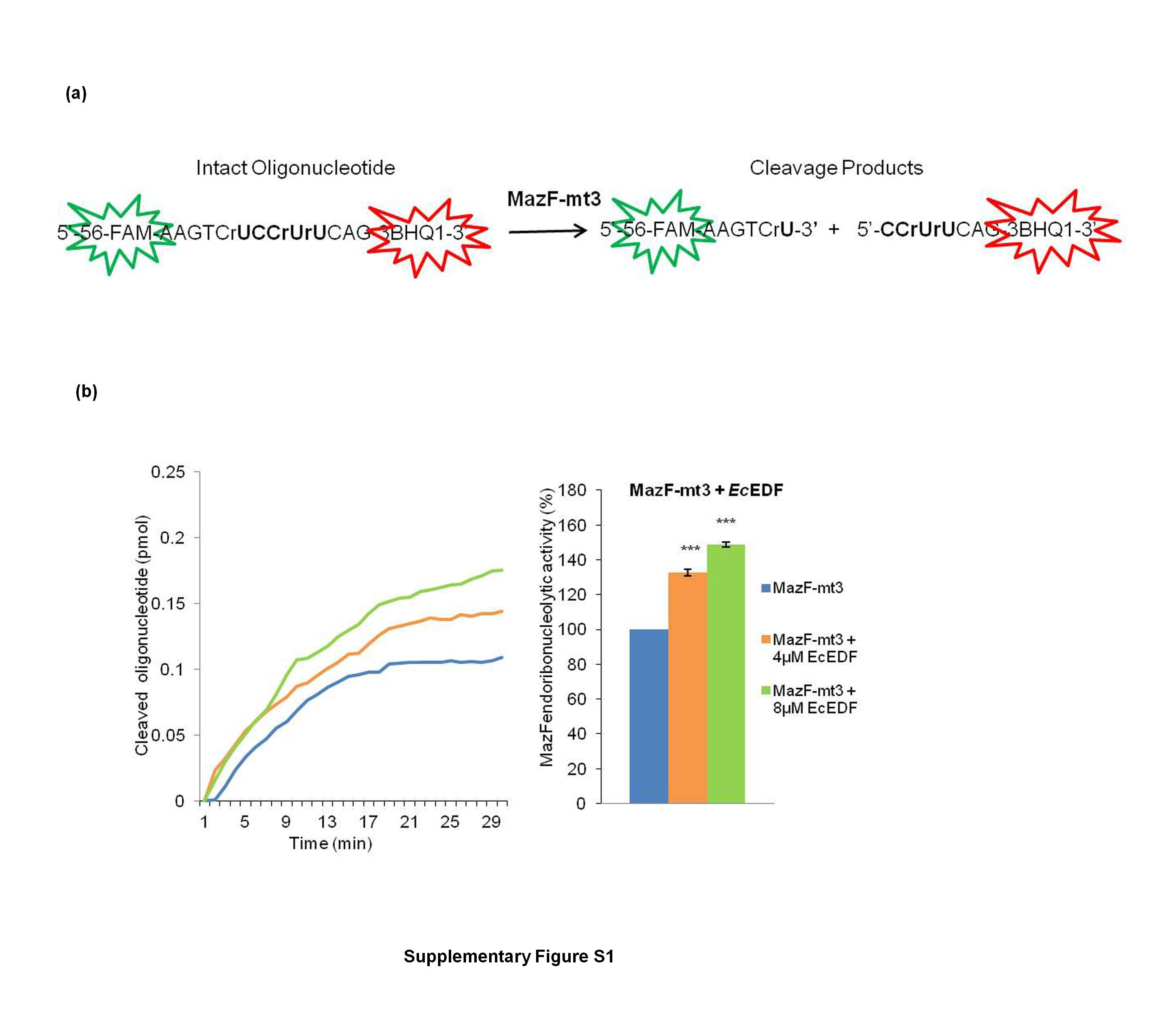

Supplement: FIG S1 [file mbo002183860sf1.tif]

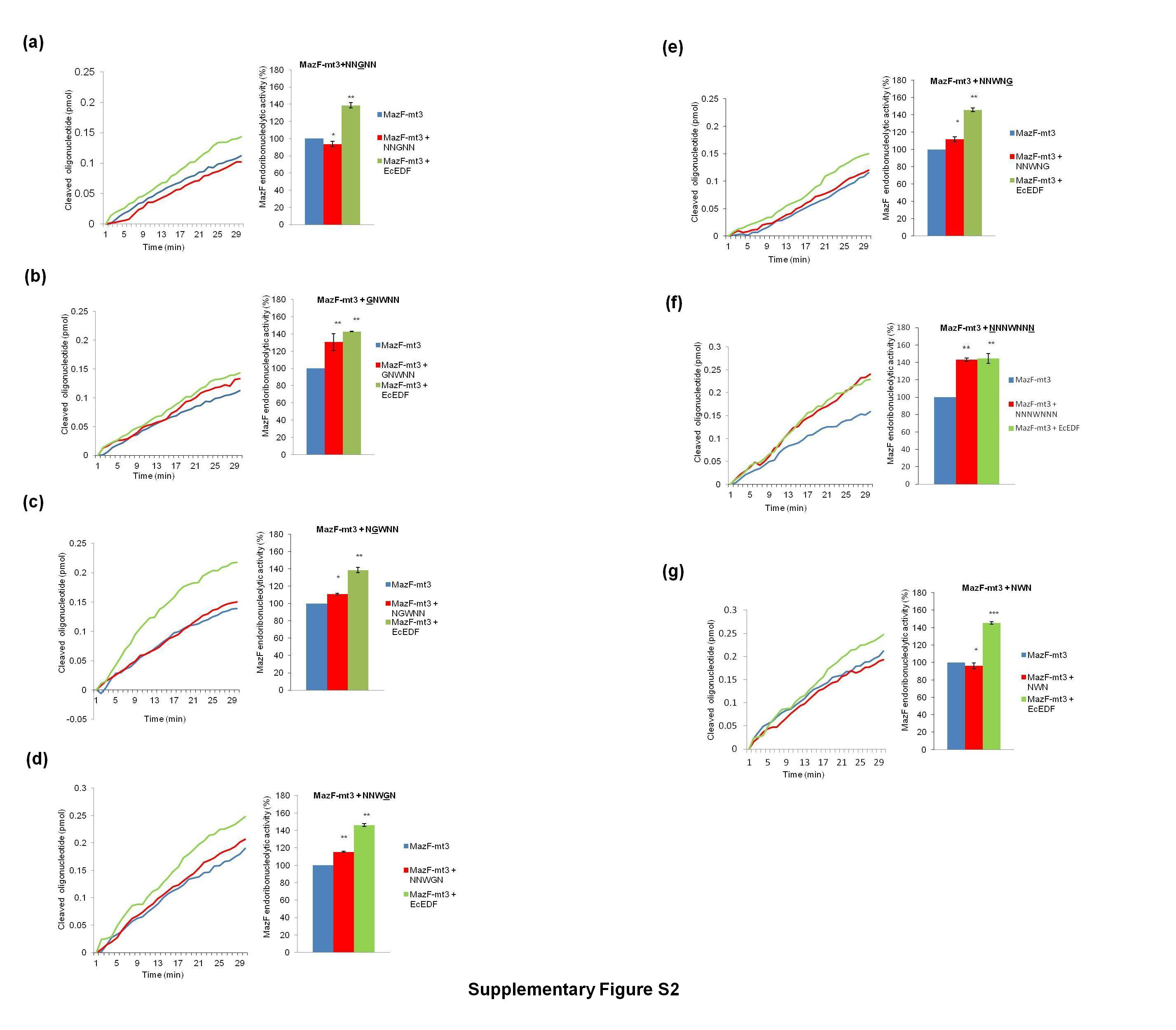

Supplement: FIG S2 [file mbo002183860sf2.tif]

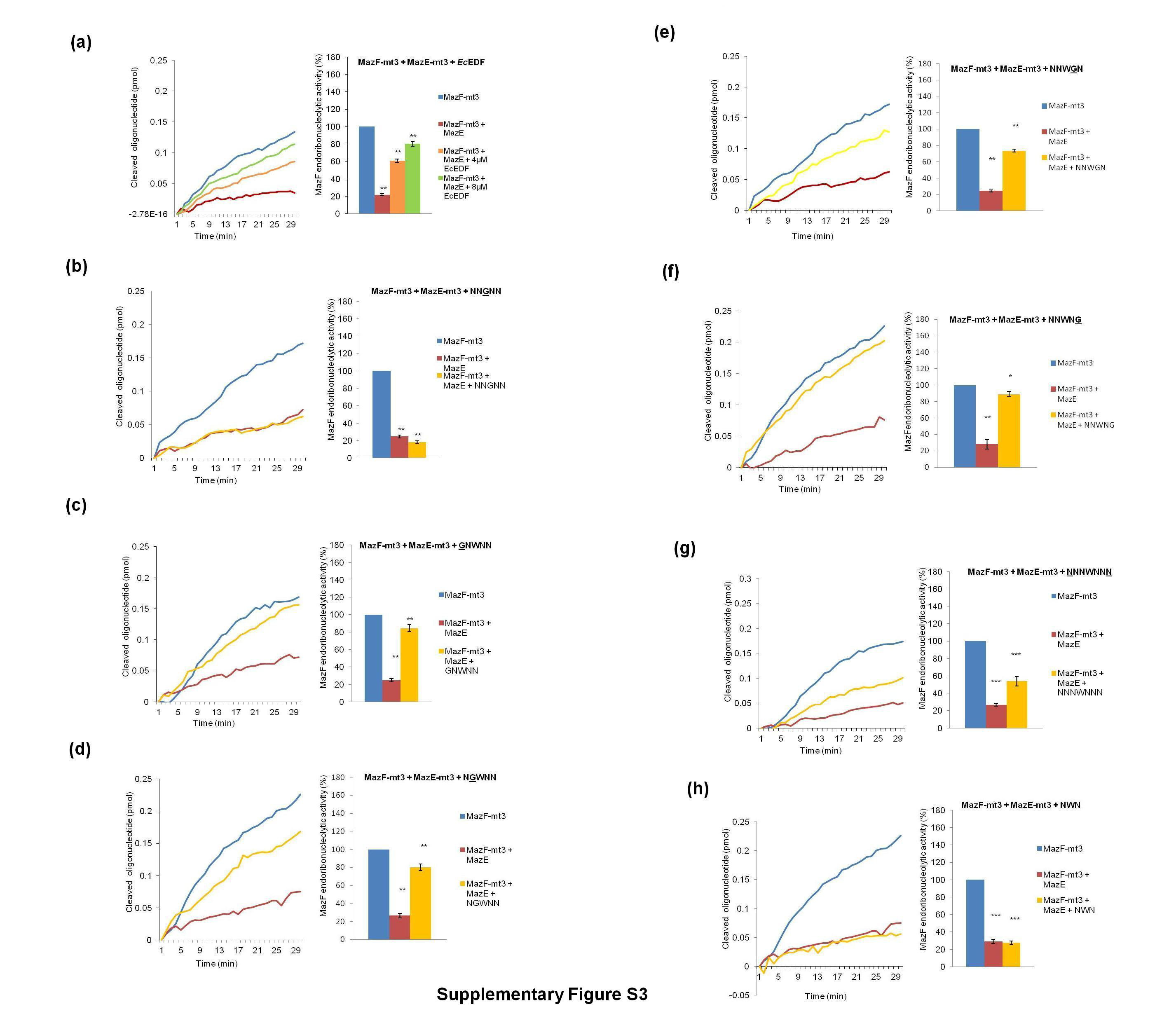

Supplement: FIG S3 [file mbo002183860sf3.tif]

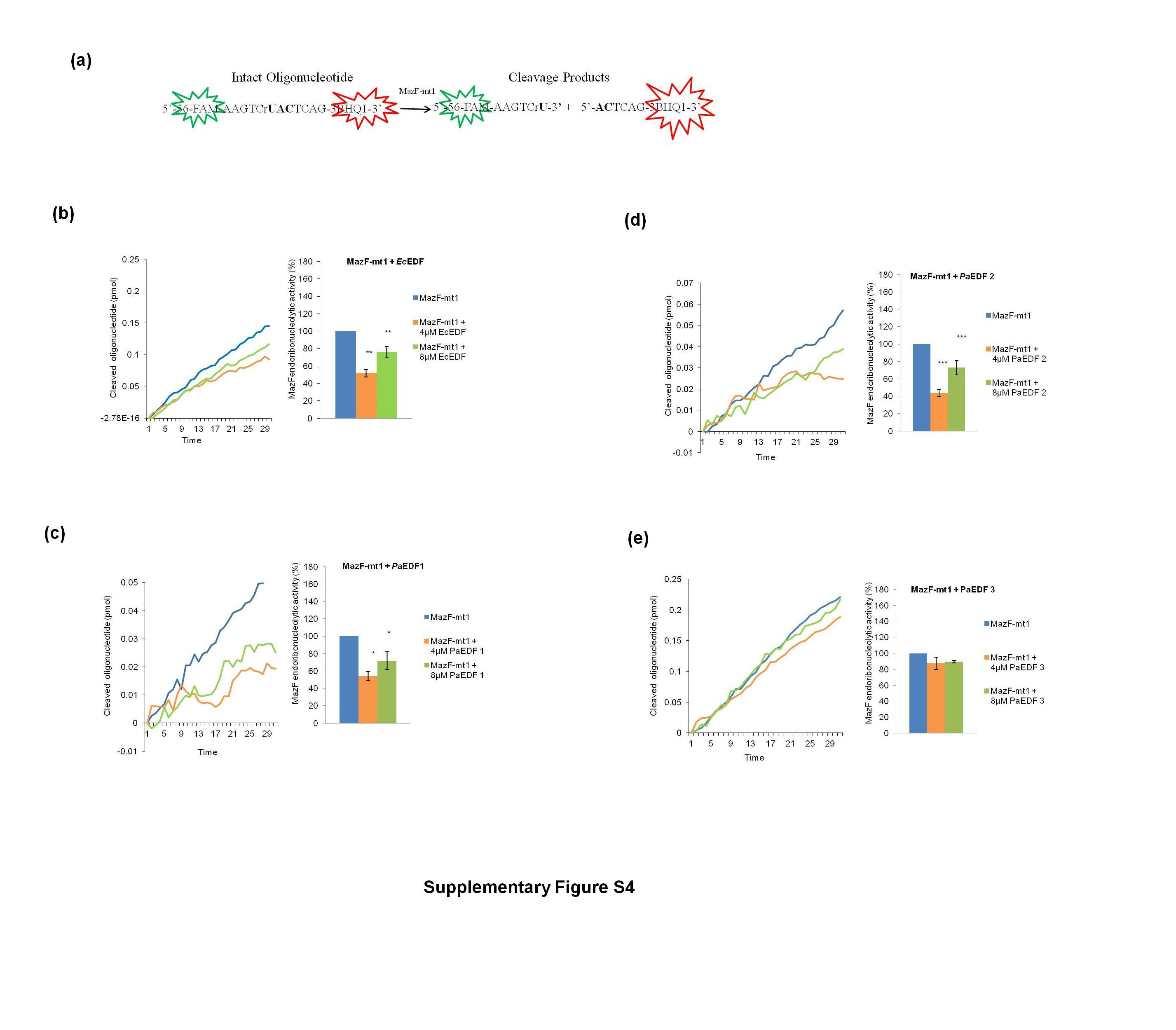

Supplement: FIG S4 [file mbo002183860sf4.tif]

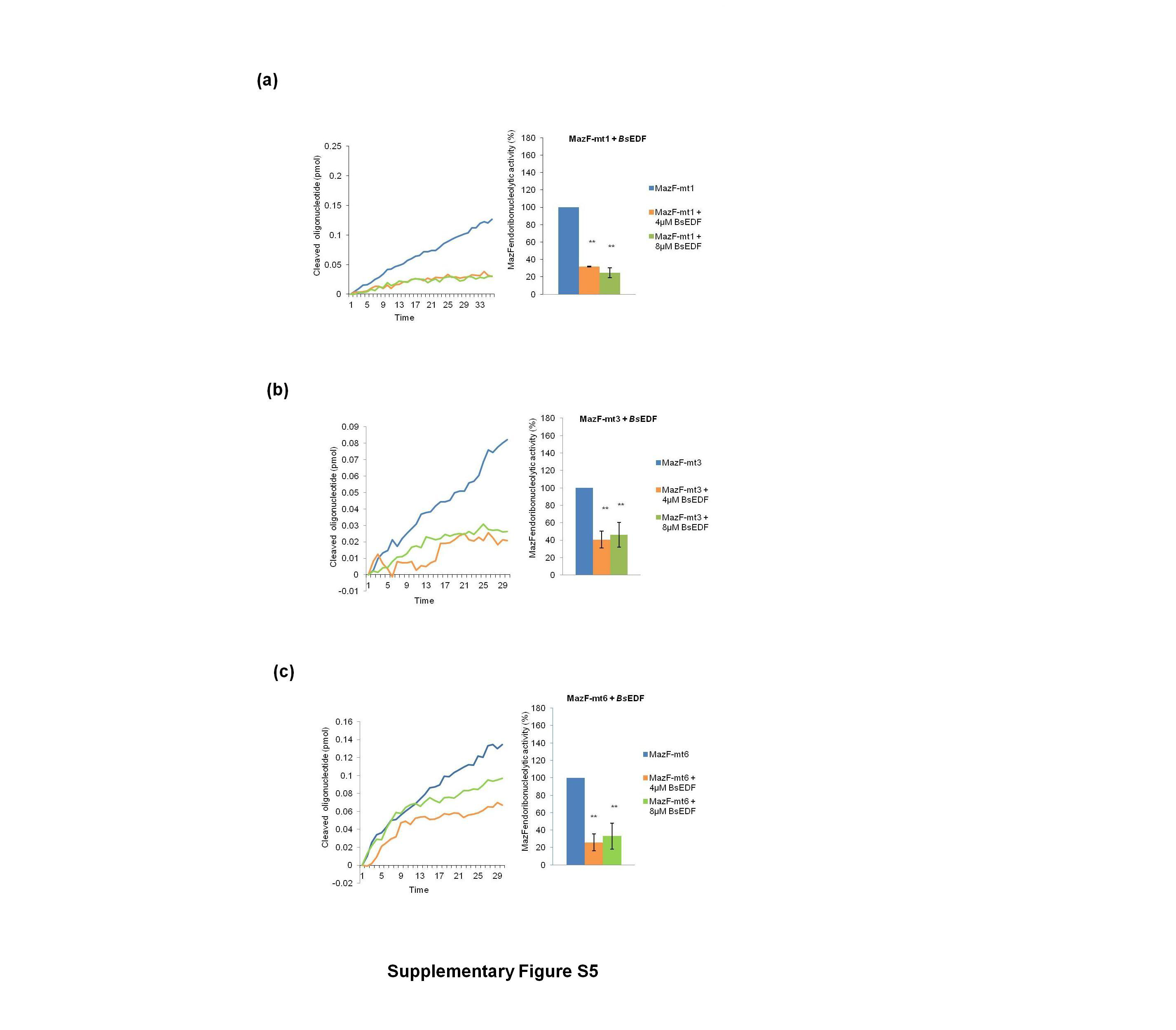

Supplement: FIG S5 [file mbo002183860sf5.tif]
