# Supplementary material for: Quorum Sensing Extracellular Death Peptides Enhance the Endoribonucleolytic Activities of Mycobacterium tuberculosis MazF Toxins
Source: mBio. 2018 May 1;9(3):e00685-18. doi: 10.1128/mBio.00685-18 (PMC5930309; doi:10.1128/mBio.00685-18)
Supplement: TEXT S1 [file mbo002183860s1.docx]

**Quorum Sensing Extracellular Death Peptides Enhance the Endoribonucleolytic Activities of *Mycobacterium tuberculosis* MazF Toxins**

**Akanksha Nigam, Sathish Kumar and Hanna Engelberg-Kulka**

**Supplementary information**

***Ec*EDF did not amplified *in vitro* endoribonucleolytic activity of *M. tuberculosis* toxin MazF-mt1.**

MazF-mt1 is another MazF of *M. tuberculosis* carrying endoribonucleolytic activity. Its target site is U↓AC. Here we asked: Will the addition of *Ec*EDF affect the activity of MazF-mt1 activity as it affected the activities of MazF-mt6 and MazF-mt3? Using a highly purified preparation of MazF-mt1, we analyzed its activity we using the continuous fluorometric assay (21). We found that the addition of neither *Ec*EDF nor any of the three *Pa*EDFs of *P. aeroginusa* led to an increase in the *in vitro* endoribonucleolytic activity of MazF-mt1. In fact, the addition of these peptides actually caused a decrease in the *in vitro* endoribonucleolytic activity of MazF-mt1 (Supplementary Figures S4a and S4b).

***B. subtilis* EDF (*Bs*EDF) did not enhance the endoribonucleolytic activity of *Mycobacterium tuberculosis* toxins MazF-mt1, MazF-mt3, MazF-mt6.**

We also asked how the presence of *Bs*EDF might affect the endoribonucleolytic activities of each of the three *M. tuberculosis* toxins MazF-mt6, MazF-mt3, and MazF-mt1. Once again, we used the fluorometric assay (21) for each of the toxins separately. Note that the addition of *Bs*EDF not only did not enhance the *in vitro* endoribonucleolytic activities of MazF-mt6, MazF-mt3, or MazF-mt1 (Fig S5a-c), but actually led to a decrease in the endoribonucleolytic activities of each of these Mycobacterial toxins.
